# Safety and efficacy of laparoscopic repeat liver resection and re-operation for liver tumor

**DOI:** 10.1038/s41598-021-89864-3

**Published:** 2021-06-02

**Authors:** Koki Takase, Takuya Sakamoto, Yutaka Takeda, Yoshiaki Ohmura, Yoshiteru Katsura, Go Shinke, Kenji Kawai, Kohei Murakami, Yoshinori Kagawa, Toru Masuzawa, Atsushi Takeno, Taishi Hata, Kohei Murata

**Affiliations:** Department of Surgery, Kansai Rosai Hospital, 3-1-69, Inabaso, Amagasaki, Hyogo 660-8511 Japan

**Keywords:** Gastroenterology, Hepatology

## Abstract

Laparoscopic liver resection (LLR) has been reported as a safe, minimally invasive, and effective surgery for the management of liver tumor. However, the efficacy and safety of laparoscopic repeat liver resection (LRLR) for recurrent liver tumor are unclear. Here, we analyzed the surgical results of LRLR. From June 2010 to May 2019, we performed 575 LLR surgeries in our department, and 454 of them underwent pure LLR for the single tumor. We classified the patients who received pure LLR for the single tumor into three groups: LRLR (n = 80), laparoscopic re-operation after previous abdominal surgery (LReOp; n = 136), and laparoscopic primary liver resection (LPLR; n = 238). We compared patient characteristics and surgical results between patients undergoing LRLR, LReOp and LPLR. We found no significant differences between LRLR and LPLR in the conversion rate to laparotomy (*p* = 0.8033), intraoperative bleeding (63.0 vs. 152.4 ml; *p* = 0.0911), or postoperative bile leakage rate (2.50 vs. 3.78%; *p* = 0.7367). We also found no significant difference in the surgical results between LReOp and LPLR. However, the number of patients undergoing the Pringle maneuver was lower in the LRLR group than the LPLR group (61.3 vs. 81.5%; *p* = 0.0004). This finding was more pronounced after open liver resection than laparoscopic liver resection (38.9 vs. 67.7%; *p* = 0.0270). The operative time was significantly longer in patients with proximity to previous cut surface than patients with no proximity to previous cut surface (307.4 vs. 235.7 min; p = 0.0201). LRLR can safely be performed with useful surgical results compared to LPLR.

## Introduction

Laparoscopic liver resection (LLR) has been reported as a safe, minimally invasive, and effective surgery for the management of liver tumor^1–10^. The indications for LLR have been expanded, and cases of laparoscopic repeat liver resection (LRLR) for hepatocellular carcinoma (HCC) and metastatic liver cancer have been increasing. However, the efficacy and safety of LRLR for recurrent liver tumor are unclear. In this study, we investigated the surgical outcomes after LRLR.

## Patients and methods

### Patients

The treatment strategy of each patient with liver cancer was selected according to Clinical Practice Guidelines for Hepatocellular Carcinoma^11^. At our institution, we used a laparoscopic approach in basically all liver resections, except liver resection with biliary or vascular reconstruction and liver resection for tumors invading the inferior vena cava.

From June 2010 to May 2019, we performed 575 LLR surgeries in our department, and 454 of them underwent pure LLR for the single tumor. We classified the patients who received pure LLR into three groups: LRLR (n = 80), re-operation after previous abdominal surgery (LReOp; n = 136), and laparoscopic primary liver resection (LPLR; n = 238). The study was approved by the Human Ethics Review Committee of Kansai Rosai Hospital (Certificate Number: 1901021) and was conducted in accordance with the Declaration of Helsinki. Each patient provided a signed informed consent.

We compared the clinical indicators of perioperative course, including patient characteristics (age, gender, Child-Pugh classification, liver damage^12^, tumor size, and diagnosis) and surgical data (extent of liver resection, difficulty score^13^, and conversion rate), and the surgical results (operative time, blood loss, postoperative morbidity, hospital stay, and laboratory data at post-operative day 1).

### Surgical procedure

During surgery, patients were supine for liver resection of a tumor in the left lobe or in the semi left lateral decubitus position for a tumor in the right lobe. The first trocar was inserted at the umbilicus by an open method. To reduce intraoperative bleeding, we tried to prepare for the Pringle maneuver in every case by encircling the hepatoduodenal ligament. However, when strong adhesion was present around the hepatoduodenal ligament, we could not use the Pringle maneuver. In cases of anatomical liver resection, we used the Glissonian pedicle approach and transected the pedicle using a surgical stapling device. Parenchymal transection was performed using an ultrasonic surgical aspirator, laparoscopic coagulation shears, and bipolar clamp coagulation system.

## Results

The patient characteristics and surgical results for the three surgical groups are given in Table 1. In the comparison between the LRLR and LPLR groups, we found no differences in gender, Child-Pugh classification, or liver damage. The rate of metastatic liver cancer was significantly higher in the LRLR group than LPLR group (22.5 vs. 4.20%; *p* < 0.0001). The tumor size was significantly smaller in LRLR group than in LPLR group (18.0 mm vs. 29.1 mm; *p* < 0.0001). The rate of Hr1 and Hr2 liver resection was significantly higher in the LPLR group (*p* = 0.0224), and the difficulty score for LLR was significantly higher in the LPLR group than LRLR group (3.8 vs. 5.0; *p* = 0.0001). In the comparison of LRLR and LPLR, the rate of Pringle maneuver preparation was significantly lower in the LRLR group (61.3 vs. 81.5%; *p* = 0.0004). We found no differences in the other surgical results, including postoperative morbidity. We also found no significant difference in the surgical results between LReOp and LPLR.

**Table 1:** Patient characteristics and surgical results of laparoscopic liver resection.

		LRLR	LReOp	LPLR	*P*
		(n = 80)	(n = 136)	(n = 238)	LRLR vs. LPLR
Age, years	Mean ± SD	72.4 ± 8.8	70.0 ± 10.7	69.5 ± 9.5	0.0169
Gender	M/F	56/24	77/59	152/86	0.3442
Child-Pugh	A/B/C	78/2/0	133/3/0	225/13/0	0.3718
Liver damage	A/B/C	58/21/1	108/28/0	184/53/1	0.3554
Diagnosis	HCC/CCC/Mets/Other	58/1/18/3	42/5/76/11	178/8/10/42	< 0.0001
Extent of liver resection	Hr0/HrS/Hr1/Hr2	63/3/6/4	85/7/22/22	155/11/47/25	0.0224
Difficulty score	L (1–3)/I (4–6)/H (7–11)^a^	41/33/6	40/53/43	71/98/69	< 0.0001
Tumor size, mm	Mean ± SD	18.0 ± 12.0	32.89 ± 61.1	29.1 ± 21.9	< 0.0001
Operative time, min	Mean ± SD	283.2 ± 131.4	333.7 ± 146.6	305.6 ± 159.6	0.2127
Blood loss, ml	Mean ± SD	63.0 ± 140.3	77.2 ± 198.0	152.4 ± 775.5	0.0906
Pringle	Possible/Impossible	49/31	110/26	194/44	0.0004
Conversion to	Assisted/HALS/Open	0/2/0	1/1/0	0/4/2	0.8033
Surgical margin	(+)/Suspicious/(−)	0/2/78	0/1/135	0/3/235	0.6029
Postoperative morbidity	Hemorrhage	0	1	0	n.s
	Biliary fistula	2	2	9	0.7367
Hospital stay, days	Mean ± SD	13.2 ± 28.8	12.1 ± 19.6	14.0 ± 19.0	0.8104
**Postoperative Laboratory Data (POD1)**					
AST, IU/l	Mean ± SD	413.7 ± 463.3	423.0 ± 320.3	462.6 ± 468.3	0.4184
ALT, IU/l	Mean ± SD	266.2 ± 308.0	297.3 ± 232.5	337.0 ± 347.4	0.1057
T-Bil, mg/dl	Mean ± SD	1.04 ± 0.55	0.91 ± 0.46	0.93 ± 0.48	0.0835
WBC, /μl	Mean ± SD	9019 ± 2948	9609.6 ± 2976	9776 ± 3069	0.0547
CRP, mg/dl	Mean ± SD	1.75 ± 1.59	2.16 ± 1.60	1.85 ± 2.12	0.6407

*HCC* hepatocellular carcinoma, *CCC* cholangiocellular carcinoma, *Mets* metastasis, *HALS* hand-assisted laparoscopic surgery, *n.s.* not significant.

^a^L: low, I: intermediate, H: high.

In the LRLR group, 62 patients underwent LLR (post-laparoscopic group), and 18 patients underwent open liver resection (post-open group) in their last surgery. The patient characteristics and surgical results of these two groups are given in Table 2. We found no differences in the patients’ characteristics except gender. Comparing the post-laparoscopic and post-open groups, the rate of Pringle maneuver preparation was significantly lower in the post-open group (67.7 vs. 38.9%; *p* = 0.0270). We found no differences in the other surgical results, including operative time (279.3 vs. 296.4 min; *p* = 0.6294), blood loss (64.4 vs. 58.2 ml; *p* = 0.8700), conversion rate, morbidity, and postoperative hospital stay.

**Table 2:** Patient characteristics and surgical results of laparoscopic repeat hepatectomy classified by the last hepatectomy.

		Post-laparoscopic group	Post-open group	*P*
		(n = 62)	(n = 18)	
Age, years	Mean ± SD	72.2 ± 9.4	72.9 ± 6.1	0.7664
Gender	M/F	40/22	16/2	0.0470
Child-Pugh	A/B/C	60/2/0	18/0/0	0.4403
Liver damage	A/B/C	47/14/1	11/7/0	0.3466
Diagnosis	HCC/CCC/Mets/Other	44/0/15/3	14/1/3/0	0.1906
Extent of liver resection	Hr0/HrS/Hr1/Hr2	52/2/4/4	15/1/2/0	0.6220
Difficulty score	L (1–3)/I (4–6)/H (7–11)^a^	30/27/5	11/6/1	0.6342
Tumor size, mm	Mean ± SD	18.9 ± 13.1	14.8 ± 6.39	0.0781
Operative time, min	Mean ± SD	279.3 ± 124.8	296.4 ± 155.3	0.6294
Blood loss, ml	Mean ± SD	64.4 ± 146.3	58.2 ± 121.0	0.8700
Pringle	Possible/Impossible	42/20	7/11	0.0270
Conversion to	Assisted/HALS/Open	1/0/0	1/0/0	0.3456
Surgical margin	(+)/Suspicious/(−)	0/2/60	0/0/18	0.4403
Postoperative morbidity	Hemorrhage	0	0	n.s
	Biliary fistula	2	0	0.4403
Hospital stay, days	Mean ± SD	14.5 ± 32.6	8.7 ± 3.2	0.4505
**Postoperative Laboratory Data (POD1)**				
AST, IU/l	Mean ± SD	391.1 ± 352.1	491.3 ± 738.0	0.4227
ALT, IU/l	Mean ± SD	281.9 ± 337.5	211.8 ± 167.4	0.3983
T-Bil, mg/dl	Mean ± SD	0.99 ± 0.49	1.22 ± 0.70	0.1291
WBC, /μl	Mean ± SD	8800 ± 2800	9772 ± 3389	0.2202
CRP, mg/dl	Mean ± SD	1.84 ± 1.63	1.42 ± 1.44	0.3184

*HCC* hepatocellular carcinoma, *CCC* cholangiocellular carcinoma, *Mets* metastasis, *HALS* hand-assisted laparoscopic surgery, *n.s.* not significant.

^a^L: low, I: intermediate, H: high.

We also evaluated the proximity of the first liver resection and second liver resection in the LRLR group. When the first and second liver resections were in the same segment or adjacent segments (lateral-medial-anterior–posterior), we classified these cases as “proximity to previous cut surface”. Other cases we classified as “no proximity to previous cut surface”. The patient characteristics and surgical results of these groups are given in Table 3. In regards to patient characteristics, the Child-Pugh classification and liver damage were worse in the no proximity to previous cut surface group (*p* = 0.0448 and *p* = 0.0328, respectively). The rate of HCC was higher in the no proximity to previous cut surface group; on the other hand, the rate of metastatic liver cancer was higher in the proximity to previous cut surface group. The operative time was significantly longer in the proximity to previous cut surface group (307.4 vs. 235.7 min; *p* = 0.0201). The rate of Pringle maneuver preparation was significantly higher in the proximity to previous cut surface group (69.8 vs. 44.4%; *p* = 0.0277). We found no differences in the other patient characteristics and surgical results, including tumor size, blood loss, conversion rate, morbidity, or postoperative hospital stay.

**Table 3:** Patient characteristics and surgical results of laparoscopic repeat liver resection classified by proximity to previous cut surface of the last hepatectomy.

		Proximity to previous Cut Surface	No Proximity to previous Cut Surface	*P*
		(n = 53)	(n = 27)	
Age, years	Mean ± SD	73.1 ± 8.8	71.1 ± 8.7	0.3504
Gender	M/F	38/15	18/9	0.6424
Child-Pugh	A/B/C	53/0/0	25/2/0	0.0448
Liver damage	A/B/C	43/10/0	15/11/1	0.0328
Diagnosis	HCC/CCC/Mets/Other	34/0/16/3	24/1/2/0	0.0276
Extent of liver resection	Hr0/HrS/Hr1/Hr2	44/1/4/4	22/2/2/0	0.3205
Difficulty score	L (1–3)/I (4–6)/H (7–11)^a^	24/23/6	17/10/0	0.1152
Tumor size, mm	Mean ± SD	17.0 ± 5.62	18.4 ± 14.1	0.5294
Operative time, min	Mean ± SD	307.4 ± 148.8	235.7 ± 67.9	0.0201
Blood loss, ml	Mean ± SD	216.5 ± 1058.9	38.1 ± 121.1	0.3872
Pringle	Possible/Impossible	37/16	12/15	0.0277
Conversion to	Assisted/HALS/Open	0/2/0	0/0/0	0.3067
Surgical margin	(+)/Suspicious/(−)	0/1/52	0/1/26	0.6226
Postoperative morbidity	Hemorrhage	0	0	n.s
	Biliary fistula	1	1	0.6226
Hospital stay, days	Mean ± SD	10.5 ± 14.5	18.5 ± 45.3	0.2421
**Postoperative Laboratory Data (POD1)**				
AST, IU/l	Mean ± SD	461.8 ± 547.0	319.1 ± 199.5	0.1943
ALT, IU/l	Mean ± SD	299.3 ± 363.3	202.9 ± 134.1	0.1921
T-Bil, mg/dl	Mean ± SD	1.02 ± 0.58	1.09 ± 0.49	0.6021
WBC, /μl	Mean ± SD	9068 ± 2858	8922 ± 3170	0.8360
CRP, mg/dl	Mean ± SD	2.01 ± 1.69	1.23 ± 1.24	0.0351

*HCC* hepatocellular carcinoma, *CCC* cholangiocellular carcinoma, *Mets* metastasis, *HALS* hand-assisted laparoscopic surgery, *n.s.* not significant.

^a^L: low, I: intermediate, H: high.

To adjust for liver resection background, we divided the LRLR and LPLR groups into low difficulty score (1–3), intermittent difficulty score (4–6), and high difficulty score (7–11) groups and compared the patients’ characteristics and surgical results (Table 4). In the low difficulty score group, patient age was significantly older with LRLR than LPLR (73.8 vs. 69.0 years; *p* = 0.0168). In the low and intermediate group, tumor size was significantly smaller in LRLR than in LPLR. In each group, the rate of metastatic liver cancer was significantly higher in the LRLR group (low: 21.9 vs. 7.0%, *p* = 0.0136; intermediate: 15.2 vs. 3.0%, *p* = 0.0074; high: 66.7 vs. 2.9%, *p* = 0.0007). We found no significant differences in the rate of Pringle maneuver preparation (low: 51.2 vs. 59.1%, *p* = 0.4356; intermediate: 69.7 vs. 85.7%, *p* = 0.0656; high: 83.3 vs. 98.6%, *p* = 0.1546). In the high difficulty score group, the hospital stay was significantly shorter for the LRLR group (11.3 vs. 22.4 days; *p* = 0.0064). Among the laboratory data (post-operative day 1), total bilirubin after LRLR was significantly higher in the low difficulty score group (1.03 vs. 0.84 g/dl; *p* = 0.034). We found no differences in the other patient characteristics or surgical results.

**Table 4:** Patient characteristics and surgical results of laparoscopic repeat liver resection classified by difficulty score.

		LRLR			LPLR			*P*		
		Low	Intermediate	High	Low	Intermediate	High	Low	Intermediate	High
		n = 41	n = 33	n = 6	n = 71	n = 98	n = 69			
Age, years	Mean ± SD	73.8 ± 8.93	70.5 ± 8.45	73.3 ± 8.61	69.0 ± 10.6	69.3 ± 8.83	70.3 ± 9.39	0.0168	0.4789	0.4550
Gender	M/F	27/14	26/7	3/3	45/26	60/38	47/22	0.8401	0.0896	0.3938
Child-Pugh	A/B/C	41/0/0	31/2/0	6/0/0	67/4/0	93/5/0	65/4/0	0.2946	0.8323	0.5444
Liver damage	A/B/C	27/14/0	25/7/1	6/0/0	53/18/0	73/24/1	58/11/0	0.3866	0.5675	0.5835
Diagnosis	HCC/CCC/Mets/Other	29/1/9/2	27/0/5/1	2/0/4/0	50/1/5/15	75/1/3/19	53/6/2/8	0.0136	0.0075	0.0007
Extent of liver resection	Hr0/HrS/Hr1/Hr2	41/0/0/0	26/2/4/1	0/1/2/3	70/0/1/0	79/2/16/1	6/9/30/24	0.4453	0.3586	0.9135
Tumor size, mm	Mean ± SD	14.9 ± 6.11	18.9 ± 10.1	31.7 ± 30.3	18.4 ± 8.63	26.6 ± 16.9	43.1 ± 29.2	0.0166	0.0023	0.4105
Operative time, min	Mean ± SD	235 ± 79	308.6 ± 140.6	475.1 ± 167.3	191.4 ± 99.6	279.2 ± 114.5	460.7 ± 144.3	0.0196	0.2304	0.8163
Blood loss, ml	Mean ± SD	41.1 ± 121.0	81.8 ± 163.3	108.3 ± 120.1	191.1 ± 56.2	67.7 ± 133.7	409.8 ± 1403.9	0.2766	0.6212	0.0909
Pringle	Possible/Impossible	21/20	23/10	5/1	42/29	84/14	68/1	0.4357	0.0656	0.1546
Conversion to	Assisted/HALS/Open	0/0/0	0/0/0	0/2/0	0/0/0	0/1/0	1/3/2	n.s	0.5602	0.4493
Surgical margin	( ±)/Suspicious/(-)	0/0/41	0/2/31	0/0/6	0/2/69	0/1/97	0/0/69	0.5317	0.1562	n.s
Postoperative morbidity	Hemorrhage	0	0	0	0	0	0	n.s	n.s	n.s
	Biliary fistula	0	2	0	2	1	6	0.5317	0.1562	0.4514
Hospital stay, days	Mean ± SD	9.2 ± 7.5	18.5 ± 43.9	11.3 ± 4.4	10,3 ± 9.7	11.2 ± 11.8	21.9 ± 29.5	0.5105	0.3503	0.0103
**Postoperative Laboratory Data (POD1)**										
AST, IU/l	Mean ± SD	283.9 ± 216.0	549.3 ± 641.3	554.3 ± 321.8	304.3 ± 523.9	410.3 ± 336.3	699.6 ± 482.9	0.7737	0.2411	0.4733
ALT, IU/l	Mean ± SD	188.5 ± 167.6	344.5 ± 420.0	365.5 ± 208.9	201.4 ± 310.9	284.5 ± 231.7	551.1 ± 418.0	0.7750	0.4389	0.2878
T-Bil, mg/dl	Mean ± SD	1.03 ± 0.48	0.92 ± 0.41	1.80 ± 1.04	0.84 ± 0.38	0.84 ± 0.43	1.15 ± 0.56	0.0346	0.3371	0.1921
WBC, /μl	Mean ± SD	9261 ± 3325	8600 ± 2449	9667 ± 2883	9114 ± 2967	9448 ± 3014	10,923 ± 2978	0.8099	0.1465	0.3239
CRP, mg/dl	Mean ± SD	1.41 ± 1.17	1.8 ± 1.606	3.68 ± 2.65	2.03 ± 2.81	1.80 ± 1.96	1.73 ± 1.41	0.1099	0.9914	0.1352

*HCC* hepatocellular carcinoma, *CCC* cholangiocellular carcinoma, *Mets* metastasis, *HALS* hand-assisted laparoscopic surgery, *n.s.* not significant.

We compared the patient characteristics and surgical results between the LRLR and LPLR groups only in HCC patients (Table 5). In the comparison between the LRLR and LPLR groups, we found no differences in gender, Child-Pugh classification, or liver damage. The age was significantly older in the LRLR group than in the LRLR group (72.8 vs 70.1; *p* = 0.029). The tumor size was significantly smaller in LRLR group than in LPLR group (15.2 mm vs. 28.9 mm; *p* < 0.0001). The operative time was significantly shorter in the LRLR group than in the LPLR group (267 min vs 323 min; *p* = 0.0065). We found no differences in the other surgical results, including postoperative morbidity.

**Table5:** Patient characteristics and surgical results of laparoscopic liver resection in HCC patients.

		LRLR	LPLR	*P*
		(n = 58)	(n = 178)	
Age, years	Mean ± SD	72.8 ± 7.8	70.1 ± 8.8	0.0299
Gender	M/F	42/16	116/62	0.3083
Child-Pugh	A/B/C	56/2/0	166/12/0	0.3565
Liver damage	A/B/C	39/18/1	130/47/1	0.5526
Extent of liver resection	Hr0/HrS/Hr1/Hr2	50/2/6/0	117/10/38/13	0.0652
Difficulty score	L (1–3)/I (4–6)/H (7–11)^a^	29/27/2	50/75/53	< 0.0001
Tumor size, mm	Mean ± SD	15.2 ± 5.52	28.9 ± 21.4	< 0.0001
Operative time, min	Mean ± SD	266.9 ± 123.1	322.5 ± 159.7	0.0065
Blood loss, ml	Mean ± SD	67.7 ± 153.4	187.9 ± 892.0	0.0867
Pringle	Possible/Impossible	33/25	158/20	< 0.0001
Conversion to	Assisted/HALS/Open	0/0/0	0/3/2	0.4351
Surgical margin	(+)/Suspicious/(−)	0/2/56	0/2/176	0.2336
Postoperative morbidity	Hemorrhage	0	0	n.s
	Biliary fistula	1	7	0.4196
Hospital stay, days	Mean ± SD	12.9 ± 31.2	14.8 ± 20.5	0.6686
**Postoperative Laboratory Data (POD1)**				
AST, IU/l	Mean ± SD	427.8 ± 523.6	515.4 ± 500.5	0.2660
ALT, IU/l	Mean ± SD	262.0 ± 343.9	366.7 ± 355.5	0.0485
T-Bil, mg/dl	Mean ± SD	1.01 ± 0.45	0.94 ± 0.50	0.3313
WBC, /μl	Mean ± SD	8769 ± 2908	9656 ± 3115	0.0503
CRP, mg/dl	Mean ± SD	1.43 ± 1.35	1.42 ± 1.30	0.9594

*HCC* hepatocellular carcinoma,* CCC* cholangiocellular carcinoma, *Mets* metastasis, *HALS* hand-assisted laparoscopic surgery, *n.s.* not significant.

^a^L: low, I: intermediate, H: high.

## Discussion

Repeat liver resection is widely performed for primary liver cancer and metastatic liver cancer^14–19^. In this study, the tumor size was significantly smaller in the LRLR group than in LPLR group. This difference may be caused by early detection of the recurrent tumors because of the routine surveillance after primary liver resection.

In this study, the rate of Pringle maneuver was significantly lower in LRLR group and this finding was more pronounced after open liver resection than laparoscopic liver resection. This difference was also found between the LRLR and LPLR groups only in HCC patients. Although whether we perform the Pringle maneuver or not is decided by surgeons, the lower rate may be caused by the difficulty of encircling the hepatoduodenal ligament because of the intra-abdominal adhesion.

Although LRLR was not commonly performed, several studies have been reported regarding the safety and issues^20–28^. Noda et al.^20^ compared 20 patients undergoing minimally invasive repeat liver resection and 48 undergoing ORLR. Minimally invasive repeat liver resection included 6 hybrid LLR patients and 14 pure LLR patients. The hybrid LLR patients underwent liver resection through a mini-laparotomy incision. The postoperative hospital stay, blood loss, and postoperative complication rate were significantly lower in the minimally invasive repeat liver resection group. They showed the safety of minimally invasive repeat liver resection, but included hybrid laparoscopic resection. In our study, we investigated the safety and efficacy of LRLR for the patients receiving pure LLR.

The use of difficulty scoring was proposed by Ban et al.^13^. The system has become widely used as an indicator of the difficulty of LLR^29^. We found a significant difference in the difficulty score between LRLR and LPLR. Therefore, we divided the LRLR and LPLR groups according to difficulty. In the low difficulty score group, the operative time was longer with LRLR. In all difficulty score groups, we found no significant difference in blood loss. The extended operative time may be caused by postoperative adhesions and liver malformation.

In repeat liver resection, many cases are technically difficult because of postoperative adhesions and liver malformation, even if they are scored as low difficulty. Kinoshita et al.^30^ analyzed 60 LRLR patients for HCC, 31 of whom underwent laparoscopic previous liver resection and 29 open previous liver resection. They reported that the predictive factors for difficult LRLR for HCC were an open approach during previous liver resection, a history of two or more previous liver resections, a history of previous major liver resection, tumor near the resected site of the previous liver resection, and intermediate or high difficulty score. In the present study, we also found that proximity to previous cut surface predicted LRLR difficulty, as the proximity to previous cut surface group had a longer operative time. The previous study analyzed LRLR only in HCC patients, whereas we investigated not only HCC, but also metastatic liver cancer and other liver tumors.

This study has some limitations. This is a retrospective, single center study. In addition, there may be some surgeon’s selection bias within the decision of operative strategy. Therefore, to show the effectiveness and safety of LRLR, more studies are needed, including multi-institutional studies and long-term observations. However, to the best of our knowledge, this study describes the largest number of LRLR surgeries at a single institution. Therefore, we showed the safety and effectiveness of LRLR.
